# A Meta-Analysis of the Impact of Mindfulness-Based Stress Reduction Therapy Intervention on Post-traumatic Stress Disorder

**DOI:** 10.7759/cureus.95793

**Published:** 2025-10-31

**Authors:** Xinmin Jiang, Minghui Zhang, Jiaqi Wang, Jing Wang, Yu Zhang

**Affiliations:** 1 College of Stomatology, Qilu Medical University, Zibo, CHN; 2 School of Stomatology, Qilu Medical University, Zibo, CHN; 3 Academic Affairs Office, School of Stomatology, Qilu Medical University, Zibo, CHN; 4 Dentistry, Qilu Medical University, Zibo, CHN

**Keywords:** depression, meta‐analysis, mindfulness‐based stress reduction, post‐traumatic stress disorder, randomized controlled tria

## Abstract

This meta-analysis was conducted to investigate the effect of mindfulness-based stress reduction (MBSR) on the treatment and improvement of traumatic stress disorder (PTSD). Up until February 6, 2025, PubMed, Web of Science, and Embase databases were searched for randomized controlled trials of MBSR-based treatment for PTSD patients. RevMan 5.4 software (The Cochrane Collaboration, London, UK) was used to conduct a meta-analysis of these trials. A total of 832 participants from nine qualified randomized controlled trials were enrolled. Meta-analysis results showed that in PTSD patients, depression scores after Mindfulness-Based Stress Reduction (MBSR) intervention were significantly lower compared with the control group (Standardized Mean Difference (SMD) = -0.25, 95% CI -0.39 to -0.10; P = 0.0006). Quality of life (SMD = 0.40, 95% CI 0.10-0.70; P = 0.008) and Five Facet Mindfulness Questionnaire (SMD = 0.41, 95% CI 0.24-0.57; P < 0.00001) scores were significantly higher than in the control group. PTSD symptoms (SMD = -0.20, 95% CI -0.31 to -0.09; P = 0.0003), various PTSD checklist scores (SMD = -1.28, 95% CI -0.33 to -0.03; P = 0.02), and clinician-administered PTSD scale scores (SMD = -0.23, 95% CI -0.39 to -0.07; P = 0.004) were also significantly reduced following MBSR intervention. MBSR can reduce depression, PTSD symptoms, and improve the quality of life and mindfulness in PTSD patients.

## Introduction and background

Post-traumatic stress disorder (PTSD) is a severe form of stress condition brought on by extreme mental stress, such as abrupt catastrophic events or natural catastrophes. PTSD can result in traumatic reliving, hypervigilance, and avoidance or numbing symptoms and is frequently seen in high-risk populations, including soldiers, survivors of concentration camps, and victims of natural disasters [1]. The Diagnostic and Statistical Manual of Mental Disorders (DSM-5) has clear diagnostic criteria for PTSD [2].

Although PTSD often manifests three months following a stressful experience, it can occasionally occur months or even years later. Precise evaluation of individuals who have gone through traumatic experiences will aid in the early detection and management of PTSD, improve the prognosis of patients, and aid in their social function rehabilitation [3]. The two primary categories of PTSD assessment instruments used worldwide are screening scales and diagnostic assessments. Of them, the PTSD Checklist (PCL) and Clinician-Administered PTSD Scale (CAPS) are used frequently [4]. The PCL is a scale for self-evaluation. PCL has been shown in numerous studies to have strong validity and reliability, as well as significant reference value for the diagnosis of PTSD [5]. CAPS is a comprehensive diagnostic tool based on DSM-IV. It is a semi-structured interview form with 30 items covering 17 core symptoms and eight related symptoms, divided into three subscales: re-experience, avoidance, and hyperarousal [6]. CAPS has emerged as the most popular standardized diagnostic assessment tool in the realm of trauma and is acknowledged as one of the gold standards for diagnosing PTSD [7].

Psychotherapy and medication are currently the primary therapies for PTSD [8]. PTSD is frequently treated with tricyclic antidepressants, monoamine oxidase inhibitors like phenelzine, and selective serotonin reuptake inhibitors [9]. Psychological treatments for PTSD include mindfulness therapy, repetitive eye movement desensitization therapy, and trauma-focused cognitive behavioral therapy [10]. Common mindfulness-based therapies include acceptance and commitment therapy (ACT), mindfulness-based pain management (MBPM), mindfulness-based cognitive therapy (MBCT), and mindfulness-based stress reduction (MBSR) [11]. One of the earliest of these is MBSR. Numerous researchers have demonstrated that MBSR helps PTSD patients with their symptoms [12]. MBSR is an approach to awareness that focuses on awareness, being present, and nonjudgment. Its goal is to empower patients to take advantage of their own capabilities and take action for their own physical and emotional well-being that others are unable to take. This approach was established by Dr. Jon Kabat-Zinn at the University of Massachusetts Medical Center’s Stress Reduction Clinic. [13]. MBSR is usually a group training course lasting eight to ten weeks. Specific methods include breathing attention, sitting meditation, body scan, and mindful yoga [14].

Recently, many systematic reviews and meta-analyses have analyzed the clinical efficacy of mindfulness-based therapy in the treatment of PTSD. Mindfulness-based intervention has a significant effect on improving the symptoms of post-traumatic stress disorder in PTSD patients. However, the studies did not distinguish between specific mindfulness-based therapies [15]. The effectiveness of mindfulness-based therapies, including MBSR and MBCT approaches, on PTSD was meta-analyzed by Haller et al [16]. The fundamental distinction between MBSR and MBCT is that MBSR focuses on mindfulness in relation to stress management and mental health, whereas MBCT primarily addresses negative emotions like anxiety and depression and preserves mental health by recognizing emotional and thought patterns [17]. A meta-analysis of MBSR treatments for PTSD was carried out by Liu et al., although they only used PCL and CAPS scale scores to examine PTSD symptoms, did not differentiate between other PTSD test scales, and chose fewer outcome indicators [18]. Thus, our objective is to investigate how MBSR intervention affects PTSD, raise outcome measures including mindfulness, depression, and quality of life, and distinguish between different PTSD scales. Through this meta-analysis, we present data supporting the use of MBSR to treat PTSD, identify key influencing aspects of MBSR intervention on PTSD, and offer references for further research.

## Review

Methods

Search Strategy

We searched the following electronic databases: PubMed, Web of Science, Embase. The final search was conducted on 6 February 2025. The search strategy used is shown in Table 1.

**Table 1:** Search strategy

Database	Date of final search	Search string
PubMed, Web of Science, Embase	6 February 2025	(“Stress Disorders, Post-Traumatic” OR “Post-Traumatic Stress Disorder” OR “Stress Disorder, Post-Traumatic” OR “Post Traumatic Stress Disorder” OR “Neuroses, Post-Traumatic” OR “Post-Traumatic Neuroses” OR “PTSD” OR “Stress Disorder, Post Traumatic” OR “Post-Traumatic Stress Disorders” OR “Post Traumatic Stress Disorders” OR “Posttraumatic Stress Disorders” OR “Posttraumatic Stress Disorder” OR “Stress Disorder, Posttraumatic” OR “Stress Disorders, Posttraumatic” OR “Neuroses, Posttraumatic” OR “Posttraumatic Neuroses” OR “Acute Post-Traumatic Stress Disorder” OR “Acute Post Traumatic Stress Disorder” OR “Chronic Post-Traumatic Stress Disorder” OR “Chronic Post Traumatic Stress Disorder” OR “Delayed Onset Post-Traumatic Stress Disorder” OR “Delayed Onset Post Traumatic Stress Disorder” OR “Moral Injury” OR “Injury, Moral” OR “Moral Injuries”) AND (“Mindfulness Based Stress Reduction” OR “Mindfulness-Based Stress Reductions” OR “Stress Reduction, Mindfulness-Based” OR “MBSR Therapy” OR “MBSR Therapies” OR “Therapy, MBSR” OR “Mindfulness-Based Stress Reduction Therapy” OR “Mindfulness Based Stress Reduction Therapy” OR “Mindfulness-Based Stress Reduction”)

Eligibility Criteria

The inclusion criteria for this study were as follows: the study design must be a randomized controlled trial; participants must be individuals who have experienced trauma, exhibit symptoms of PTSD, and are either clinically diagnosed according to DSM-IV or DSM-5 criteria, or meet PTSD thresholds based on self-reported scales; the experimental group must receive Mindfulness-Based Stress Reduction (MBSR), while the control group should receive conventional treatment or an alternative intervention; outcome measures must include depression, PTSD Checklist scores, the Clinician-Administered PTSD Scale, quality of life, and the Five Facet Mindfulness Questionnaire. Studies were excluded if they were non-randomized controlled trials; if patients were not clearly diagnosed with PTSD; if the intervention used was not MBSR; if the full text was unavailable; or in cases of duplicate publications.

Literature Screening and Data Extraction Method

In order to exclude the literature that clearly did not satisfy the inclusion criteria, two researchers separately searched the database, reviewed the abstracts and titles of the literature, and then studied the complete texts of the literature that potentially fit the requirements. After screening, the two researchers double-checked the literature, and the third researcher made the decision if there was a disagreement. First author, publication time, region, sample size, age, intervention measures, intervention time, primary outcome indicators, etc. were all included in the final literature extraction material.

Literature Quality Evaluation

The Cochrane Collaboration Risk of Bias tool [16,17] was used to assess the included studies that examined study quality in seven areas of trial design (random sequence generation, allocation concealment, blinding of participants and personnel, blinding of outcome assessment, incomplete outcome data, selective reporting and other bias), ranking each area as high, low, or unclear for risk of bias.

Statistical Analysis

RevMan 5.4 software (The Cochrane Collaboration, London, UK) was used for statistical analysis. Standardized mean difference (SMD) was used as the effect index for quantitative data. Each effect index provided a 95% confidence interval (CI). P < 0.05 indicated statistical significance.. P‐value and I^2^ statistics were used to assess statistical heterogeneity. Of the I^2^, 25% was interpreted as low heterogeneity, 50% as moderate heterogeneity, and 75% as high heterogeneity.

Results

Study Selection

**Figure 1:**
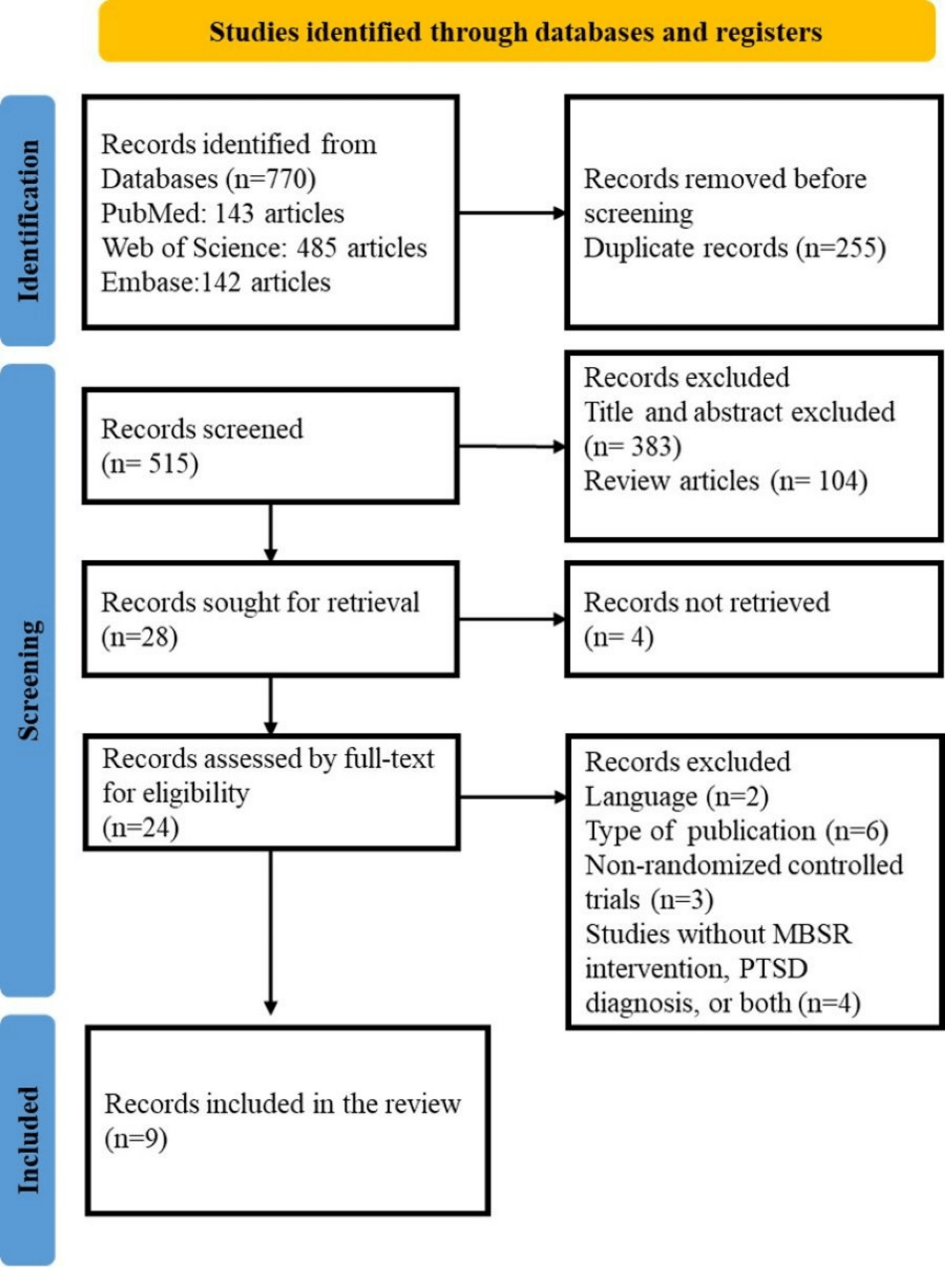
PRISMA flow chart of study selection

After retrieving 770 records in total, 255 were eliminated because they were duplicates. Titles and abstracts that were deemed unnecessary were eliminated from 104 reviews and 383 articles. Nine publications satisfied the inclusion criteria after non-English language articles, non-RCTs, conference papers, and other ineligible studies were excluded. The article inclusion and exclusion process is shown in Figure 1.

Description of Included Studies

Nine randomized controlled studies were included, involving 832 participants. The age range of the subjects included in the studies was 18-64 years old. The detailed information is shown in Table 2. All patients were diagnosed with post-traumatic stress. MBSR was the main intervention method in the experimental group. Among the intervention methods in the control group, five studies used present-centered group therapy (PCGT), three used treatment as usual (TAU), and one study used wellness control (teach participants how to improve their physical and emotional health and wellbeing).

**Table 2:** Description of individual studies Abbreviations: CAPS, Clinician-Administered PTSD Scale; MBSR, mindfulness-based stress reduction; PCGT, present-centered group therapy; PCL, PTSD Checklist; PHQ, Patient Health Questionnaire; FFMQ, Five-Facet Mindfulness Questionnaire; TAU, treatment as usual; BRUMS, Brunel Mood Scale (Inventory of Mood Status); SCID-I, Structured Clinical Interview for DSM-IV; BDI-II, Beck Depression Inventory-2; WHOQOL-BREF, World Health Organization Quality of Life–Brief.

Study	Region	N (each condition)	Age (year)	Male (%)	Intervention	Intervention time	Measures
Davis et al., 2019 [19]	USA	96/95	51.7 (10.9)/ 51.0 (11.4)	84	MBSR/PCGT	8-week	CAPS, PCL
Kang et al., 2022 [20]	USA	47/51	58.6 (10.4)/ 59.4 (9.3)	86	MBSR/PCGT	8-week	CAPS, PCL, PHQ-9
Kearney et al., 2013 [21]	USA	25/22	52 (3.4)/ 52(11.7)	79	MBSR/TAU	8-week	PCL, PHQ-9, FFMQ
Polusny et al., 2015 [22]	USA	58/58	57.6 (10.4)/ 59.4 (9.2)	84	MBSR/PCGT	8-week	CAPS, PCL, PHQ-9, WHOQOL-BREF, FFMQ
Shapira et al., 2022 [23]	USA	104/106	55(12)	84	MBSR/PCGT	8-week	CAPS, PCL, PHQ-9
Omidi et al., 2013 [24]	Iran	31/31	39 – 59	100	MBSR/TAU	8-week	BRUMS
Omidi et al., 2018 [25]	Iran	31/31	35 – 49	100	MBSR+MBCT/TAU	8-week	SCID-I, BDI-II
Bremner et al., 2017 [26]	USA	9/8	34 (7)/ 35 (10)	100	MBSR/PCGT	8-week	CAPS, FFMQ
Gallegos et al., 2020 [27]	USA	19/10	18-64	0	MBSR/Wellness Control	8-week	PCL

Quality Evaluation of Literature

**Figure 2:**
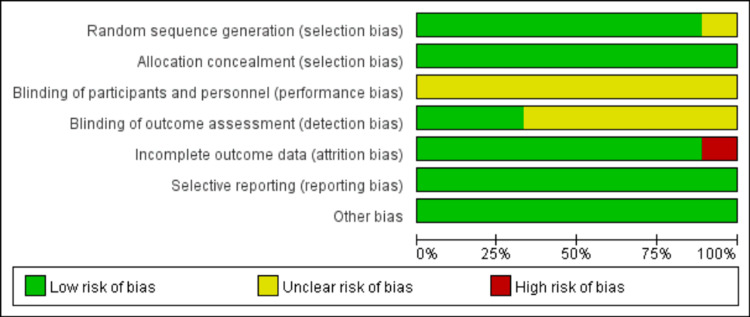
Quality assessment in included studies

**Figure 3:**
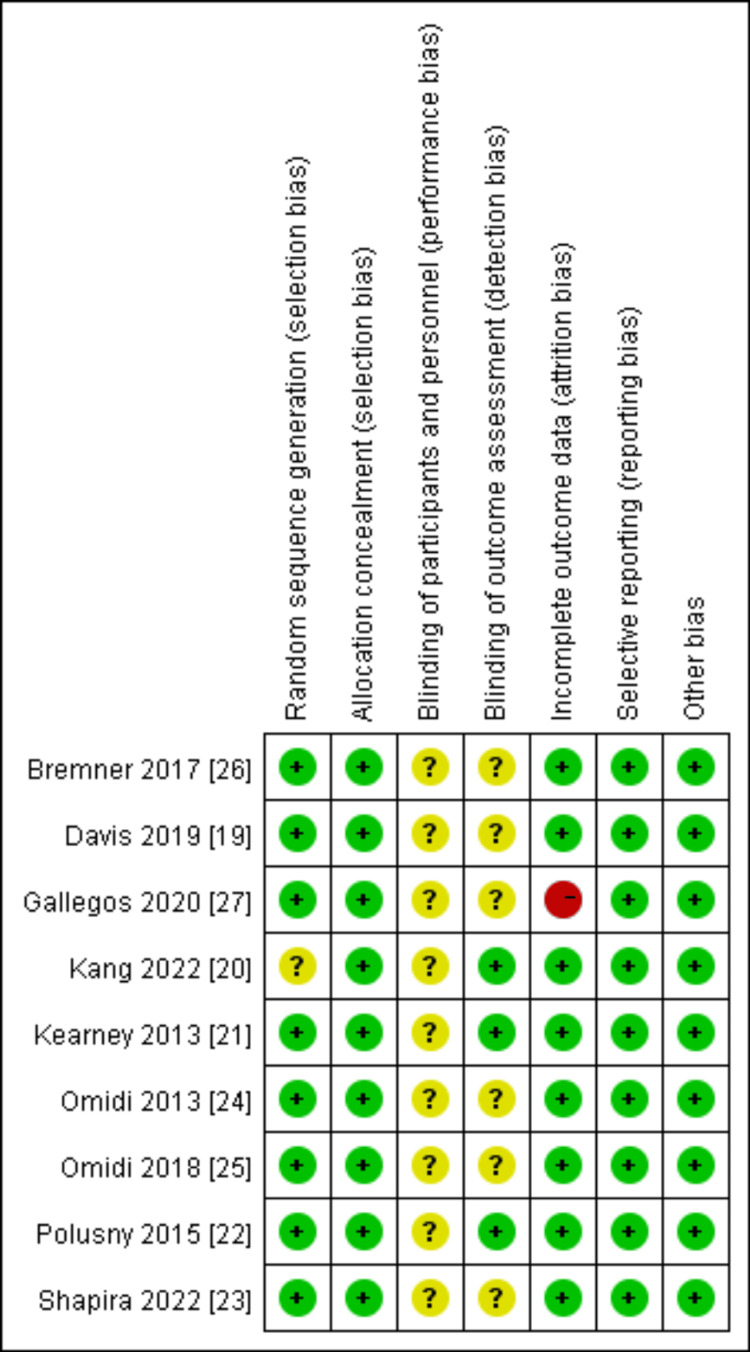
The summary of quality assessment in included studies

The quality of all included literature was evaluated according to the Cochrane 5.1.0 Manual for Systematic Reviews. The evaluation results are shown in Figures 2-3.

Effects of MBSR Intervention on Depression in PTSD

**Figure 4:**
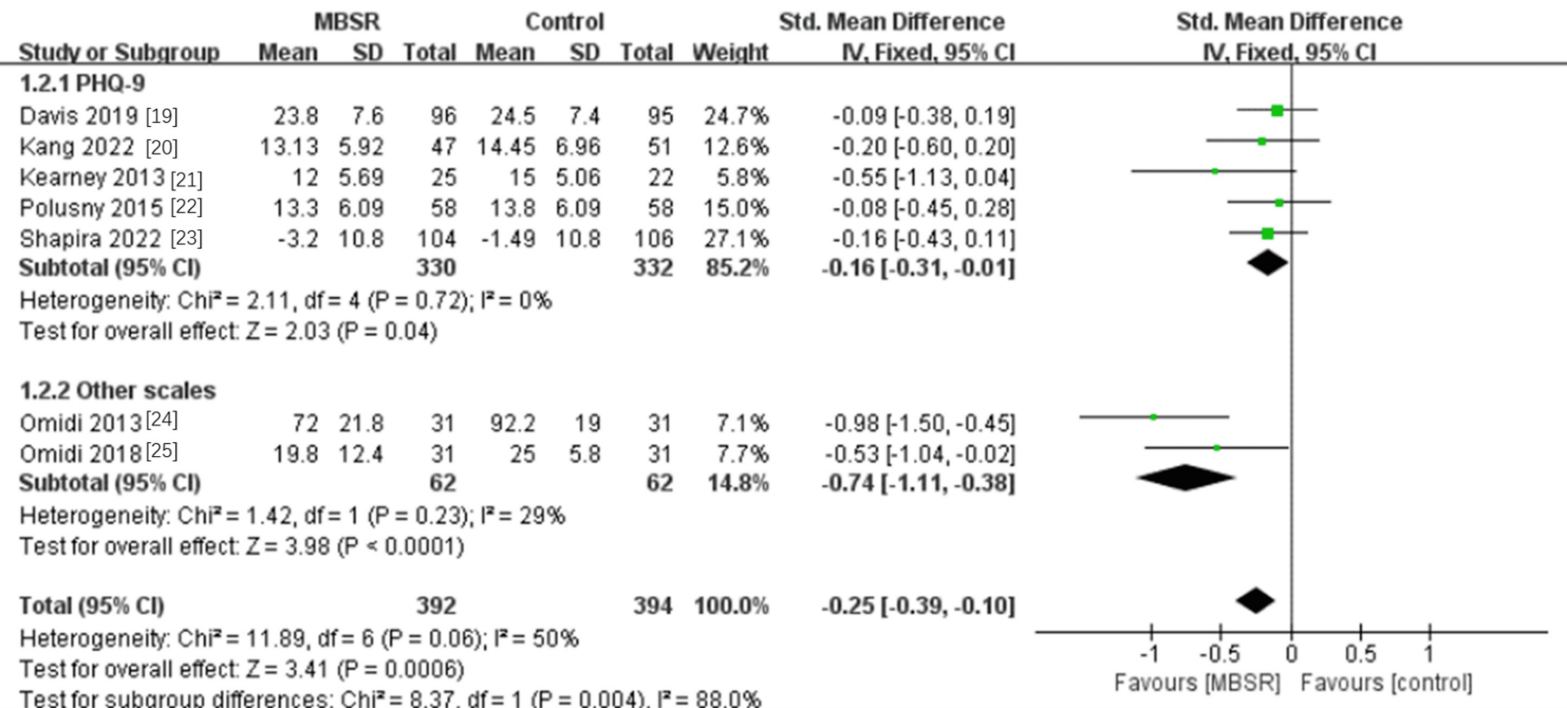
A meta-analysis of the effects of MBSR on depression in patients with PTSD MBSR, mindfulness-based stress reduction; PTSD: post-traumatic stress disorder

Of the 786 participants in the seven included studies, five used the Patient Health Questionnaire-9 (PHQ-9) to measure depression [19-23]. In this subgroup research, there was no heterogeneity (P = 0.72, I^2^ = 0%). Utilizing a fixed-effects model, the findings demonstrated that MBSR could lower depression as measured by the PHQ-9, with a statistically significant combined effect (SMD= -0.16, 95% CI -0.31 to -0.01; P = 0.04). Two studies used different measures to measure depression [24,25]. Mild heterogeneity was evident in the results (P = 0.23, I^2^ = 29%). The findings demonstrated that MBSR had a statistically significant combined effect on reduced depression as measured by other measures (SMD= -0.74, 95% CI -1.11 to -0.38; P <0.0001). Overall, MBSR can significantly reduce depression in patients with PTSD (SMD= -0.25, 95% CI -0.39 to -0.10; P = 0.0006) (see Figure 4).

Effect of MBSR Intervention on the Mindfulness Ability of Patients With PTSD

**Figure 5:**
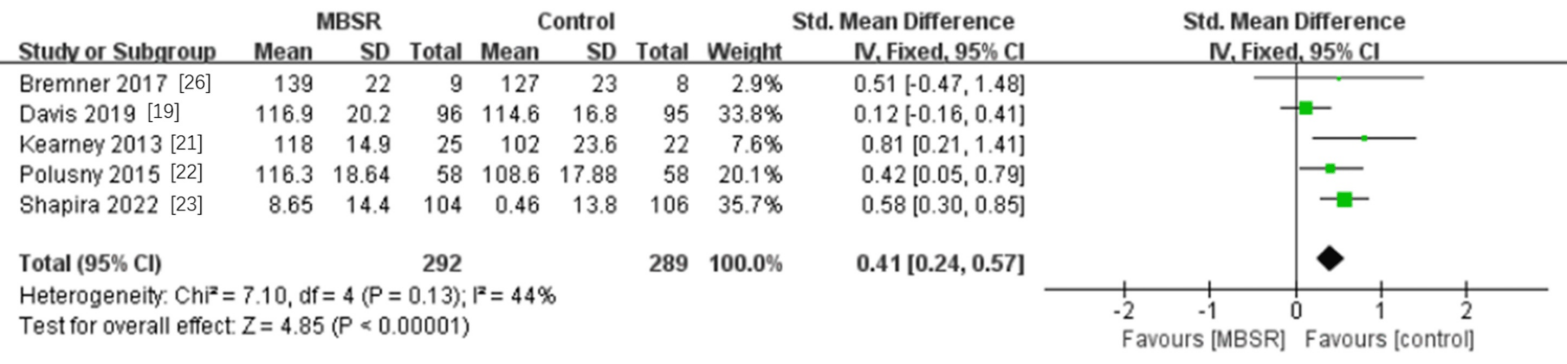
A meta-analysis of the effects of MBSR on mindfulness in patients with PTSD MBSR, mindfulness-based stress reduction; PTSD: post-traumatic stress disorder

The five included studies measured the mindfulness ability of 581 participants using the Five Facet Mindfulness Questionnaire scale [19,21-23,26], and the results were slightly heterogeneous (P = 0.13, I^2^ = 44%). Using a fixed-effects model, the results showed that MBSR can significantly improve the mindfulness ability of PTSD patients, and the combined effect was statistically significant (SMD=0.41, 95% CI 0.24 to 0.57; P< 0.00001) (see Figure 5).

Effect of MBSR Intervention on the Quality of Life of Patients With PTSD

**Figure 6:**
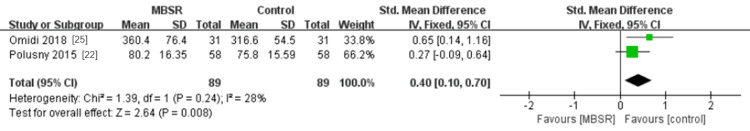
Meta-analysis of the effect of MBSR on the quality of life of patients with PTSD MBSR, mindfulness-based stress reduction; PTSD: post-traumatic stress disorder

Two studies measured the quality of life of 178 patients [22,25], and the results were slightly heterogeneous (P = 0.24, I^2^ = 28%). Using a fixed-effects model, the results showed that MBSR can significantly improve the quality of life of PTSD patients, and the combined effect was statistically significant (SMD=0.40, 95% CI 0.10 to 0.70; P=0.008) (see Figure 6).

Effects of MBSR Intervention on PTSD Symptoms

**Figure 7:**
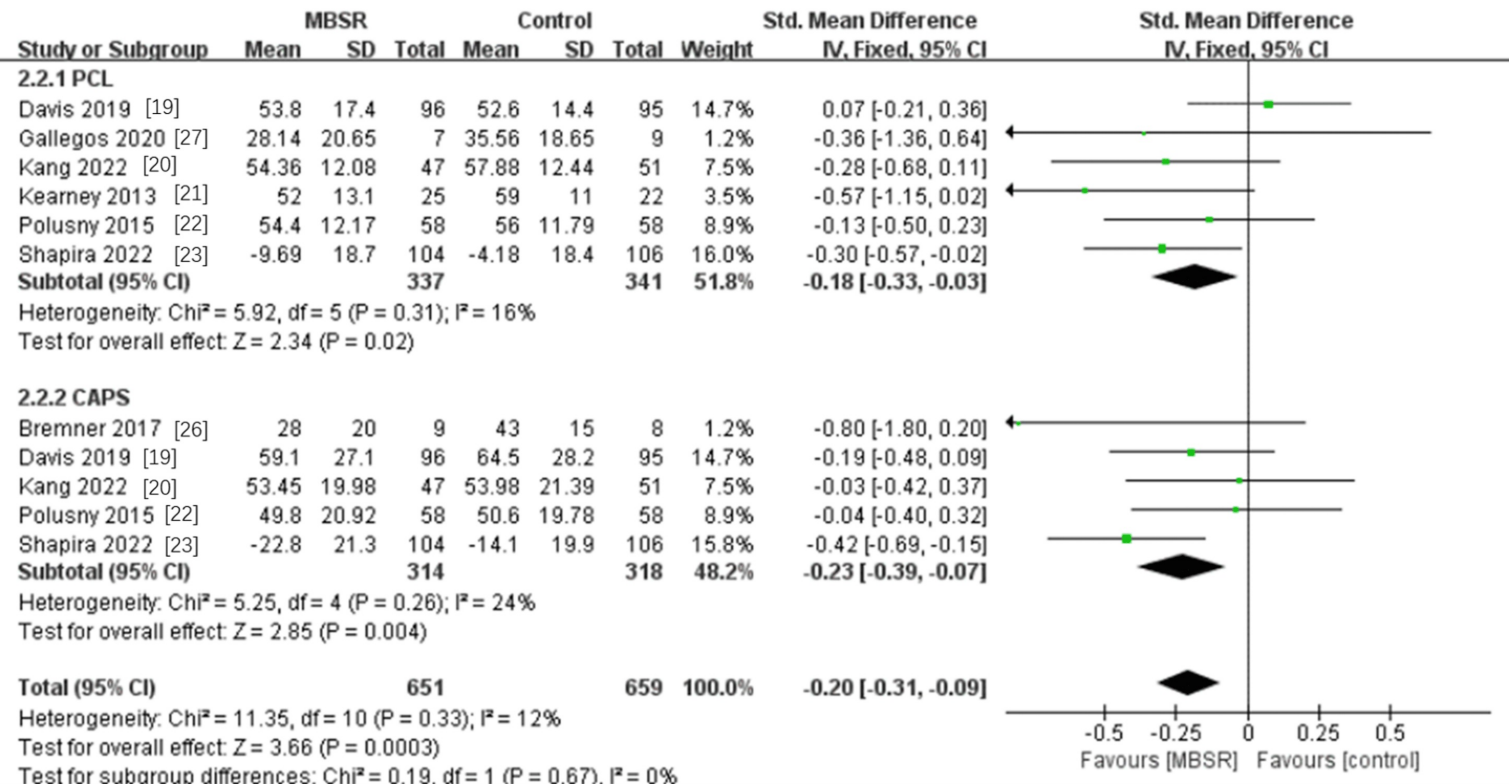
A meta-analysis of the effects of MBSR on PTSD symptoms MBSR, mindfulness-based stress reduction; PTSD: post-traumatic stress disorder

Six studies were included in the subgroup measuring PTSD symptoms by the PCL scale, measuring 678 participants [19-23,27]. The results of this subgroup were slightly heterogeneous (P= 0.31, I^2^ = 16%). Using a fixed-effects model, the results showed that MBSR could reduce PTSD symptoms assessed by the PCL scale, and the combined effect was statistically significant SMD=-0.18, 95% CI -0.33 to -0.03; P = 0.02). The subset that assessed 632 participants' PTSD symptoms using the CAPS scale comprised five research studies [19,20,22,23,26]. Mild heterogeneity was evident in the results (P = 0.26, I^2^ = 24%). The findings demonstrated that MBSR also had a statistically significant moderating effect on PTSD symptoms as measured by the CAPS scale (SMD= -0.23, 95% CI -0.39 to -0.07; P <0.004). Overall, MBSR can significantly reduce PTSD symptoms in patients with PTSD (SMD= -0.20, 95% CI -0.31 to -0.09; P = 0.0003) (see Figure 7).

Discussion

This meta-analysis details how MBSR helps patients with PTSD. This study examined the effects of MBSR on depression, mindfulness, quality of life, and PTSD symptoms in PTSD patients by analyzing data from nine randomized trials involving 832 participants. The outcomes of this experiment were found to be reasonably stable after sensitivity analysis. According to preliminary findings, MBSR can treat depression in PTSD patients, enhance their quality of life and mindfulness, and lessen their symptoms of PTSD. For analysis, the impact of MBSR on depression was separated into two subgroups: PHQ-9 and other measures. Additionally, we separated the impact on PTSD symptoms into PCL and CAPS subgroups for analysis. We discovered that MBSR considerably reduced the symptoms of PTSD and depression. This meta-analysis examines successful outcome markers and offers evidence in favor of MBSR treatment interventions for PTSD patients.

Although meta-analyses of MBSR intervention in PTSD patients have been done by other researchers, outcome variables including depression and mindfulness skill were not the main emphasis. They made no distinctions across detection scales and solely examined PTSD symptoms. Using a meta-analysis, Liu et al. discovered that the MBSR intervention group was more successful than the control group at reducing PTSD symptoms (Hedges' g = 0.461). There was no statistical difference between the two groups; however, the active control group's Hedge's g was 0.447, and the inactive control group's was 0.514 [18]. In their thorough examination of PTSD symptoms, Liu et al. made a distinction between the active and control groups. Our study distinguished between PTSD detection scales and found that the scores of the PCL (-0.28; -0.33 to -0.03; P = 0.04) and CAPS (-0.23; -0.39 to -0.07; P = 0.004) scales decreased and symptoms improved after MBSR intervention. This experimental result complements the experimental results of Liu et al., and also provides a basis for the MBSR intervention PDST symptom selection scale.

The effects of mindfulness therapy on PTSD patients have been meta-analyzed by other researchers. There is no distinction made between different mindfulness techniques in mindfulness therapy. The mindfulness of PTSD patients was evaluated by Harper et al. using the FFMQ and the Mindfulness Attention Awareness Scale (MAAS). Post-traumatic stress disorder and total mindfulness were found to be strongly correlated (r+ = -0.39, 95% CI -0.47 to - 0.30) [28]. In a meta-analysis, Hopwood et al. discovered that mindfulness-based treatment was successful in reducing PTSD symptoms when compared to a control condition (Hedges' g = - 0.44) [29]. The finding that MBSR improves PTSD symptoms is consistent with the above research trends, indicating that MBSR has certain value in clinical application as a common method of mindfulness therapy.

Huang et al. found that cognitive behavioral therapy (CBT) and mindfulness-based interventions were effective for both depression and generalized anxiety disorder (GAD) [30]. Through meta-analysis, this study came to the conclusion that MBSR can lessen the level of depression in PTSD patients. This study was split into two subgroups based on different scales: two studies used BDI-II (-0.74, 95% CI -1.11 to -0.38; P <0.0001), and five used PHQ (-0.16, 95% CI -0.31 to -0.01; P = 0.04). When the two subgroups were combined, it was found that MBSR could lower depression in PTSD patients (-0.25, 95% CI -0.39 to -0.10; P = 0.0006). Chi et al. investigated the effects of MBSR on depression in adolescents and young adults, and the experimental results showed that mindfulness-based stress reduction therapy was effective in reducing depressive symptoms (Hedges 'g = -0.45) [31]. These studies suggest that MBSR can reduce levels of depression in negative psychological problems such as PTSD.

This study also conducted a meta-analysis of MBSR's effect on mindfulness ability and quality of life. Mindfulness ability was mainly judged by FFMQ scale scores, and the analysis results showed that MBSR could significantly improve the mindfulness ability of PTSD patients (0.41, 95% CI 0.24 to 0.57; P< 0.00001). For the study of MBSR improving quality of life in PTSD patients, the commonly used scale to judge quality of life is WHOQOL-BREF [32]. Our analysis showed that MBSR improved the quality of life in PTSD patients (0.40, 95% CI 0.10 to 0.70; P=0.008). Although there are only a few studies included, the results of this study can also provide a data reference, which also indicates that there are few studies on the quality of life of PTSD patients by MBSR, and also provides ideas for follow-up studies.

The patient group may be a major factor in the small number of studies that were included in this analysis. PTSD brought on by war circumstances accounts for the majority of these included literature, while patients with PTSD brought on by other causes hardly ever have many samples in real life. Consequently, it also implies that in order to gather more information, thorough research on PTSD brought on by a variety of reasons might be carried out. This analysis also has the advantage of being able to evaluate the efficacy of intervention techniques intuitively across all of the included randomized controlled experiments. After sensitivity analysis, the experimental results are comparatively steady, and the study's heterogeneity is minimal. Furthermore, the study's outcome indicators are really extensive, and the most prevalent PTSD and depression symptoms in PTSD patients are examined on many scales, which has some reference value for subsequent research. The findings of this study also imply that several detection techniques may be used in follow-up research to thoroughly assess the impact of MBSR as an intervention on PTSD patients.

Limitations

This research has several limitations. The target population is the first one. In the target demographic included in this study, war is the primary cause of PTSD, and the results are not general because there are more men than women in this cohort. The ensuing follow-up period varies significantly, despite the MBSR intervention time being very consistent. Follow-up indicators cannot be included in accordance with the unified follow-up period, and the combined effect of follow-up is lacking. Research indicators in the supplied references are somewhat dispersed, and many outcome indicators cannot able to be analyzed together. Furthermore, only English-language literature was included; foreign-language literature was excluded.

Future Research Directions

Mindfulness-based stress reduction has a long history as a common means of intervention and treatment of negative psychological symptoms. This study found that MBSR has a significant therapeutic effect on depression, quality of life, mindfulness, and PTSD symptoms in PTSD patients. However, there are few studies on MBSR intervention for PTSD. This may be because there are many causes of PTSD, and it is difficult to gather patients with PTSD caused by the same cause for research. Therefore, we can uniformly intervene in PTSD patients caused by different causes and observe the effect of MBSR on PTSD. We can refer to the outcome indicators of this study, expand the research indicators, and measure re-experience, avoidance, and hyperarousal in the CAPS scale to improve the research on the effect of MBSR on PTSD.

## Conclusions

Using a meta-analysis, this study examined how MBSR affected PTSD patients. The following outcome indications were chosen for analysis: depression, quality of life, mindfulness and PTSD symptoms. Regardless of the PHQ-9 or other measures, studies have shown that MBSR can lower depression in PTSD patients while also enhancing their quality of life and mindfulness. Additionally, two PCL and CAPS scale subgroups were examined in this study. MBSR has been found to help PTSD sufferers with their symptoms.

## References

[1] Altawil MA, El-Asam A, Khadaroo A (2023). Impact of chronic war trauma exposure on PTSD diagnosis from 2006-2021: a longitudinal study in Palestine. Middle East Curr Psychiatry.

[2] Maheux A, Price M (2015). Investigation of the relation between PTSD symptoms and self-compassion: comparison across DSM IV and DSM 5 PTSD symptom clusters. Self Identity.

[3] Pilla P, Le JY, Lay P, Tiong J, Osier N (2021). What is PTSD? Diagnosis, treatment, and challenges. Front Young Minds.

[4] Mueser KT, Salyers MP, Rosenberg SD, Ford JD, Fox L, Carty P (2001). Psychometric evaluation of trauma and posttraumatic stress disorder assessments in persons with severe mental illness. Psychol Assess.

[5] Ibrahim H, Ertl V, Catani C, Ismail AA, Neuner F (2018). The validity of Posttraumatic Stress Disorder Checklist for DSM-5 (PCL-5) as screening instrument with Kurdish and Arab displaced populations living in the Kurdistan region of Iraq. BMC Psychiatry.

[6] Weathers FW, Bovin MJ, Lee DJ (2018). The Clinician-Administered PTSD Scale for DSM-5 (CAPS-5): Development and initial psychometric evaluation in military veterans. Psychol Assess.

[7] Lechner-Meichsner F, Steil R (2021). A clinician rating to diagnose CPTSD according to ICD-11 and to evaluate CPTSD symptom severity: Complex PTSD Item Set additional to the CAPS (COPISAC). Eur J Psychotraumatol.

[8] Petrakis IL, Simpson TL (2017). Posttraumatic stress disorder and alcohol use disorder: a critical review of pharmacologic treatments. Alcohol Clin Exp Res.

[9] Lancaster CL, Teeters JB, Gros DF, Back SE (2016). Posttraumatic stress disorder: Overview of evidence-based assessment and treatment. J Clin Med.

[10] Baas MA, van Pampus MG, Braam L, Stramrood CA, de Jongh A (2020). The effects of PTSD treatment during pregnancy: systematic review and case study. Eur J Psychotraumatol.

[11] Pérez-Fernández JI, Salaberria K, Ruiz de Ocenda Á (2022). Mindfulness-based pain management (MBPM) for chronic pain: a randomized clinical trial. Mindfulness.

[12] Cáceres Videla CE, Cáceres Melillo RC (2023). Mindfulness-based cognitive therapy (MBCT) and mindfulness-based stress reduction (MBSR) in the treatment of post-traumatic stress disorder (PTSD): a literature review. Salud Mental.

[13] Rosenbaum E (2021). Practice of mindfulness: Helping self and others. Humanist Psychol.

[14] Allen ES, Evans S, Wyka K (2021). Making the most of mindfulness-based stress reduction: participant feedback reveals strengths and challenges. Cogn Behav Pract.

[15] Ramachandran HJ, Bin Mahmud MS, Rajendran P, Jiang Y, Cheng L, Wang W (2023). Effectiveness of mindfulness-based interventions on psychological well-being, burnout and post-traumatic stress disorder among nurses: A systematic review and meta-analysis. J Clin Nurs.

[16] Haller H, Höxtermann M, Voiss P, Dobos G, Cramer H (2019). The effectiveness and safety of mindfulness-based interventions for posttraumatic stress disorder - a systematic review and meta-analysis. Adv Integr Med.

[17] Marchand WR (2012). Mindfulness-based stress reduction, mindfulness-based cognitive therapy, and Zen meditation for depression, anxiety, pain, and psychological distress. J Psychiatr Pract.

[18] Liu Q, Zhu J, Zhang W (2022). The efficacy of mindfulness-based stress reduction intervention 3 for post-traumatic stress disorder (PTSD) symptoms in patients with PTSD: A meta-analysis of four randomized controlled trials. Stress Health.

[19] Davis LL, Whetsell C, Hamner MB (2019). A multisite randomized controlled trial of mindfulness-based stress reduction in the treatment of posttraumatic stress disorder. Psychiatr Res Clin Pract.

[20] Kang SS, Sponheim SR, Lim KO (2022). Interoception underlies therapeutic effects of mindfulness meditation for posttraumatic stress disorder: a randomized clinical trial. Biol Psychiatry Cogn Neurosci Neuroimaging.

[21] Kearney DJ, McDermott K, Malte C, Martinez M, Simpson TL (2013). Effects of participation in a mindfulness program for veterans with posttraumatic stress disorder: a randomized controlled pilot study. J Clin Psychol.

[22] Polusny MA, Erbes CR, Thuras P (2015). Mindfulness-based stress reduction for posttraumatic stress disorder among veterans a randomized clinical trial. JAMA.

[23] Shapira I, Richman J, Pace TW (2022). Biomarker response to mindfulness intervention in veterans diagnosed with post-traumatic stress disorder. Mindfulness (N Y).

[24] Omidi A, Mohammadi A, Zargar F, Akbari H (2013). Efficacy of mindfulness-based stress reduction on mood States of veterans with post-traumatic stress disorder. Arch Trauma Res.

[25] Omidi A, Hamidian S (2018). Effectiveness of a combined mindfulness-based cognitive therapy and mindfulness-based stress reduction intervention on depression symptoms and quality of life in a group of Iranian veterans with posttraumatic stress disorder. Iran J Psychiatry Behav Sci.

[26] Bremner JD, Mishra S, Campanella C (2017). A pilot study of the effects of mindfulness-based stress reduction on post-traumatic stress disorder symptoms and brain response to traumatic reminders of combat in Operation Enduring Freedom/Operation Iraqi Freedom combat veterans with post-traumatic stress disorder. Front Psychiatry.

[27] Gallegos AM, Heffner KL, Cerulli C, Luck P, McGuinness S, Pigeon WR (2020). Effects of mindfulness training on posttraumatic stress symptoms from a community-based pilot clinical trial among survivors of intimate partner violence. Psychol Trauma.

[28] Harper L, Jones A, Goodwin L, Gillespie S (2022). Association between trait mindfulness and symptoms of post-traumatic stress: A meta-analysis. J Psychiatr Res.

[29] Hopwood TL, Schutte NS (2017). A meta-analytic investigation of the impact of mindfulness-based interventions on post traumatic stress. Clin Psychol Rev.

[30] Huang J, Nigatu YT, Smail-Crevier R, Zhang X, Wang J (2018). Interventions for common mental health problems among university and college students: A systematic review and meta-analysis of randomized controlled trials. J Psychiatr Res.

[31] Chi X, Bo A, Liu T, Zhang P, Chi I (2018). Effects of mindfulness-based stress reduction on depression in adolescents and young adults: A systematic review and meta-analysis. Front Psychol.

[32] Almarabheh A, Salah AB, Alghamdi M (2023). Validity and reliability of the WHOQOL-BREF in the measurement of the quality of life of Sickle disease patients in Bahrain. Front Psychol.

